# Ingenane Diterpenoids from *Euphorbia peplus*: Structure Elucidation and Autophagic Flux Activation Activity

**DOI:** 10.3390/molecules31091388

**Published:** 2026-04-23

**Authors:** Jiajia Wan, Qingyun Lu, Zifei Xu, Xiaojiang Hao, Rongcan Luo, Yingtong Di

**Affiliations:** 1 State Key Laboratory of Phytochemistry and Natural Medicines, Kunming Institute of Botany, Chinese Academy of Sciences, Kunming 650201, China; wanjiajia@mail.kib.ac.cn (J.W.); m19184437531@163.com (Q.L.); xuzifei@mail.kib.ac.cn (Z.X.); haoxj@mail.kib.ac.cn (X.H.); 2 University of Chinese Academy of Sciences, Beijing 100049, China; 3 Yunnan Characteristic Plant Extraction Laboratory, Kunming 650106, China; 4 Gansu Key Laboratory of Biomonitoring and Bioremediation for Environmental Pollution, Ministry of Education Key Laboratory of Cell Activities and Stress Adaptations, School of Life Sciences, Lanzhou University, Lanzhou 730099, China

**Keywords:** *Euphorbia peplus*, ingenane diterpenoid, autophagic flux

## Abstract

Autophagy dysfunction is implicated in the pathogenesis of Alzheimer’s disease (AD), and enhancing autophagic flux has been proposed as a potential strategy for addressing neurodegenerative diseases. To expand the structural diversity of ingenol esters and systematically evaluate their autophagic flux activation activity, a systematic phytochemical investigation of ingenane diterpenoids from *Euphorbia peplus* was conducted. A total of 13 ingenane-type compounds were isolated and identified, including two previously undescribed compounds, euphingenol A and B (**1**–**2**), together with 11 known analogs (**3**–**13**). Their structures were elucidated by extensive spectroscopic analyses (HRESIMS and NMR) and comparison with literature data. The compounds were evaluated for their bioactivity with flow cytometry in assays of autophagic flux in HM Cherry-GFP-LC3 (human microglia cells stably expressing the tandem monomeric mCherry-GFP-tagged LC3) cells. 17-*O*-benzoyl-20-deoxyingenol (**3**) significantly activated autophagic flux at concentrations of 10 μM and 40 μM, while euphingenol A (**1**) induced a dose-dependent increase, with structure-activity relationship analysis indicating that C-17 acylation enhances this bioactivity. These findings suggest that compound **3** warrants further investigation as a potential modulator of autophagic flux, possibly through binding to PKCδ (protein kinase C), with relevance to autophagy-related neurodegenerative conditions.

## 1. Introduction

Macroautophagy (herein referred to as autophagy) is an important intracellular clearance route for intracytoplasmic aggregate-prone proteins, certain pathogens, and dysfunctional organelles such as mitochondria [1]. This dynamic process, known as the autophagy–lysosomal pathway (ALP), involves the formation of autophagosomes, their fusion with lysosomes to form autolysosomes, and the subsequent breakdown of sequestered materials within the acidic lysosomal lumen [2]. Autophagy dysfunction has been implicated in the pathogenesis of various neurodegenerative diseases, including Alzheimer’s disease (AD) [3]. In AD brains, impaired neuronal autophagy leads to the accumulation of various autophagic vacuoles, such as autophagosomes and autolysosomes, within dystrophic neurites. This accumulation reflects disrupted autophagic flux and decreased degradative efficiency [4]. The impairment often results from disruption of key components within the ALP. For instance, the peroxisomal ACAA1 p.N299S mutation has been shown to promote AD pathology by disrupting the ALP [5]. Conversely, proactive intervention strategies have demonstrated therapeutic potential. An FDA-approved (Food and Drug Administration) lipid-lowering agent, primarily used for treating hyperlipidemia, has been reported to effectively reduce Aβ pathology through activation of the ALP, suggesting that targeting the autophagy pathway may represent an effective approach for intervening in AD progression [6]. Notably, the functional status of the ALP is typically assessed by monitoring autophagic flux, with enhanced autophagic flux considered a key functional readout of ALP activation [7]. Given the critical role of restoring autophagic flux in neuroprotection, as highlighted by these research advances, enhancing autophagic flux has been proposed as a potential strategy for addressing neurodegenerative diseases, including AD [7].

The genus Euphorbia has garnered considerable attention due to its production of structurally diverse diterpenoids [8]. Among these, jatrophane, lathyrane, ingenane, and tigliane-type diterpenoids have become focal points in natural product research owing to their significant pharmacological activities, including antitumor [9], anti-inflammatory [10,11], and multidrug resistance reversal effects [12]. Despite these advances, investigations into the bioactive properties of Euphorbia diterpenoids have continued to expand, revealing new therapeutic possibilities. Notably, certain abietane diterpenoids have been reported to exert anti-AD effects by targeting PPARγ and upregulating ABCA1 expression [13]. Furthermore, accumulating evidence from our laboratory and others has demonstrated that jatrophane diterpenoids possess the ability to activate the lysosomal–autophagy pathway [14]. Specifically, these compounds have been shown to enhance autophagic flux [15,16,17], facilitating the clearance of aggregation-prone proteins and dysfunctional organelles. In our previous studies, 5-angeloyloxy-ingena-1,6-dien-9-on-3*β*,4*β*-diol (HEP14, **5**) isolated from *Euphorbia peplus* and its analogs were found to induce lysosome biogenesis and promote Aβ clearance in mouse brains, suggesting that Euphorbia diterpenoids may exert neuroprotective effects through autophagy regulation [18]. Additionally, we also observed that di-substituted or perihydroxyl-masked 20-deoxyingenol esters retain the capacity to induce autophagy [19]. These cumulative findings underscore the therapeutic potential of ingenane derivatives as autophagy modulators. Consequently, there remains a need to expand the structural diversity of ingenane derivatives and systematically evaluate their autophagic flux activation activity.

Research on the metabolites of Euphorbiaceae plants has exhibited a marked species-specific concentration. Among the reported patented metabolites, 56% (*n* = 16) are derived from the temperate annual herb *E. peplus* [20]. This species, traditionally used as a medicinal plant, has been employed in the treatment of skin diseases, asthma, and cancer, with its latex and diterpenoid constituents considered the primary bioactive components [21]. Given this context, *E. peplus* was selected as the target species for further exploration of ingenane-type diterpenoids and assessment of their effects on autophagic flux. A total of 13 ingenane-type compounds were isolated and identified, including two previously undescribed compounds, euphingenol A and B (**1**–**2**), together with 11 known analogs. Euphingenol A and B (**1**–**2**) were elucidated using extensive nuclear magnetic resonance (NMR), circular dichroism (CD), ultraviolet–visible spectroscopy (UV), infrared spectroscopy (IR) and high-resolution electrospray ionization mass spectrometry (HRESIMS) experiments. Moreover, 17-*O*-benzoyl-20-deoxyingenol (**3**) significantly activated autophagic flux in the preliminary screening, highlighting its promise for further investigation as a modulator of autophagy dysfunction implicated in neurodegenerative diseases.

## 2. Results and Discussion

### 2.1. Structure Elucidation

**Figure 1:**
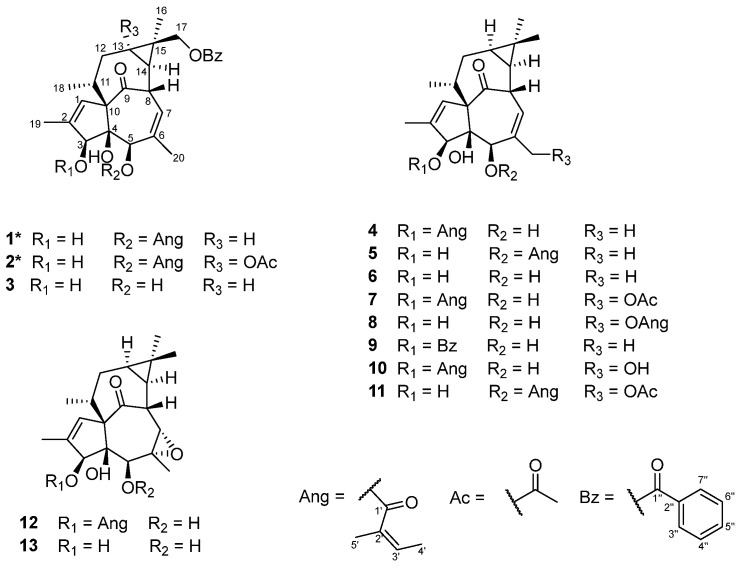
Structures of compounds **1**–**13**. Compounds with numbers followed by an asterisk (*) are previously undescribed compounds.

**Figure 2:**
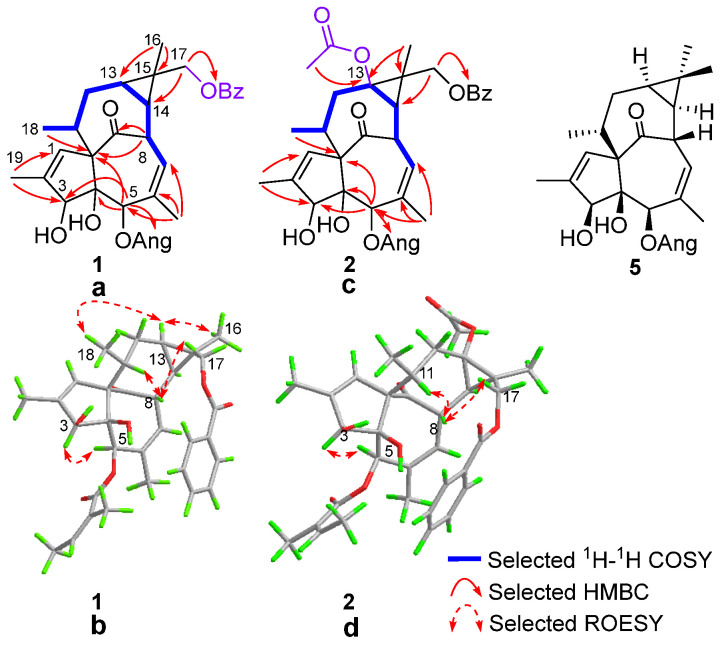
^1^H–^1^H COSY, key HMBC, and key ROESY correlations of compounds **1**–**2**. The (**a**,**c**) show the ^1^H–^1^H COSY and key HMBC correlations of compounds **1** and **2**; (**b**,**d**) show the key ROESY correlations of compounds **1** and **2**.

Compound **1** (euphingenol A) was obtained as a yellowish amorphous powder. Its optical activity was determined, with ${\left[\alpha \right]}_{D}^{24}$ +25.20 (*c* 0.15, MeOH), and the CD spectrum exhibited Cotton effects at *λ* (∆*ε*) 195 (−26.5), 204 (0.3), and 224 (−2.8), supporting the absolute configuration. Its molecular formula was established as C_32_H_38_O_7_ by HRESIMS, which displayed a sodium adduct ion peak at *m*/*z* 557.2510 [M + Na]^+^ (calcd. 557.2510), corresponding to 14 degrees of unsaturation. The ^1^H and ^13^C NMR data (Table 1) revealed diagnostic signals for a keto group [*δ*_C_ 206.1 (C-9)], an angeloyl group [*δ*_H_ 6.16 (m, H-3′), 1.98 (d, *J* = 7.3 Hz, H-4′), and 1.94 (m, H-5′); *δ*_C_ 167.1 (C-1′), 127.0 (C-2′), 140.2 (C-3′), 16.0 (C-4′), and 20.7 (C-5′)], a benzoyloxy group [*δ*_H_ 8.05 (d, *J* = 8.4 Hz, H-3″, H-7″), 7.45 (t, *J* = 7.8 Hz, H-4″, H-6″), and 7.56 (t, *J* = 7.4 Hz, H-5″); *δ*_C_ 130.4 (C-2″), 129.6× 2 (C-3″, C-7″), 128.4× 2 (C-4″, C-6″), 133.0 (C-5″), and 166.9 (C-1″)], and two trisubstituted double bonds [*δ*_H_ 5.97 (d, *J* = 1.5 Hz, H-1) and 5.85 (dt, *J* = 7.4 Hz, 1.8 Hz, H-7); *δ*_C_ 129.5 (C-1), 139.6 (C-2), 135.3 (C-6), and 125.0 (C-7)]. In addition to the functional groups described above, two hydroxyl groups were inferred from the molecular formula and are located at C-3 (*δ*_C_ 80.3) and C-4 (*δ*_C_ 85.2). These functionalities account for nine of the 14 indices of hydrogen deficiency, indicating that compound **1** contains four additional rings (Figure 1). The planar structure of **1** was established through comprehensive analysis of ^1^H–^1^H COSY and HMBC data (Figure 2a). Key HMBC correlations were observed from H_3_-18 (*δ*_H_ 17.1) to C-10 (*δ*_C_ 72.7); H_3_-19 (*δ*_H_ 1.83) to C-1 (*δ*_C_ 129.5), and C-2 (*δ*_C_ 139.6); H-5 (*δ*_H_ 5.26) to C-3 (*δ*_C_ 80.3), C-4 (*δ*_C_ 85.2), and C-10 (*δ*_C_ 72.7); H_3_-20 (*δ*_H_ 1.5) to C-5 (*δ*_C_ 76.8), C-6 (*δ*_C_ 135.3), and C-7 (*δ*_C_ 125.0); H-8 (*δ*_H_ 4.42) to C-10 (*δ*_C_ 72.7); H_3_-16 (*δ*_H_ 4.59, 4.48) to C-13 (*δ*_C_ 23.9), and C-15 (*δ*_C_ 27.9); H-17 (*δ*_H_ 1.22) to C-14 (*δ*_C_ 24.1). These data reveal that **1** possesses an ingenane-type diterpenoid skeleton similar to that of 5-angeloyloxy-ingena-1,6-dien-9-on-3*β*,4*β*-diol (**5**) [22], differing by the presence of a benzoyloxy group and oxymethylene signals in **1** and the absence of a methyl signal in **5**. The angeloyl group was confirmed by HMBC correlations from H-5′ (*δ*_H_ 1.94) to C-1′ (*δ*_C_ 167.1) and C-2′ (*δ*_C_ 127.0), and its attachment at C-5 (*δ*_C_ 76.8) was established by the HMBC correlation from H-5 (*δ*_H_ 5.26) to C-1′ [22]. Furthermore, the benzoyloxy group was located at C-17 (*δ*_C_ 24.6) based on the HMBC correlation between the oxymethylene protons H_2_-17 (*δ*_H_ 1.22) and the ester carbonyl carbon C-1″ (*δ*_C_ 166.9) of the benzoyloxy moiety (Figure 2a). This assignment is consistent with the X-ray structure of a related ingenane diterpenoid that also bears a benzoyloxy group at C-17 [23], whereas an acetyl group at the same position has been reported in another study [24].

The relative configuration of **1** was determined by ROESY analysis and coupling constant evaluation (Figure 2b). The ROESY cross-peak between H-17 and H-8 indicates a *β*-orientation for C-17. Based on biosynthetic considerations, H-4 was assigned an *α*-orientation, and Me-18 a *β*-orientation. ROESY correlations between Me-16/H-13 and H-13/Me-18 suggest that H-13, Me-16, and Me-18 are co-facial and *β*-oriented. In contrast, correlations between H-3/H-5 and H-8/H-11 indicate *α*-orientations for H-3, H-5, H-8, and H-11. The relative configurations at the chiral centers were determined to be identical to those of compound **5**, as supported by ROESY data and the close similarity of their ^13^C NMR chemical shifts (Supplementary Material Figures S1–S10). Accordingly, the structure of compound **1** was established as 5-angelate-17-benzoyl-20-deoxyingenol (euphingenol A) (Figure 1).

Compound **2** (euphingenol B) was obtained as a pale yellow amorphous solid. Its optical activity was revealed by ${\left[\alpha \right]}_{D}^{24}$ +40.45 (*c* 0.05, MeOH), and the CD spectrum displayed Cotton effects at *λ* (∆*ε*) 196 (22.7) and 224 (−3.0), consistent with the established absolute configuration. Its molecular formula was established as C_34_H_40_O_9_ by HRESIMS, which displayed a sodium adduct ion peak at *m*/*z* 615.2566 [M + Na]^+^ (calcd. 615.2566), corresponding to 15 degrees of unsaturation. The NMR data of **2** (Table 1) show close similarity to those of **1**, indicating that **2** also possesses an ingenane-type diterpenoid skeleton. However, distinct differences were observed: compound **2** exhibited additional signals for an acetyloxy group [*δ*_C_ 21.2, 170.8; *δ*_H_ 2.01 (s, *J* = 2.01 Hz)] and an oxygenated quaternary carbon (*δ*_C_ 68.7), while the methine signal corresponding to C-13 in compound **1** (*δ*_C_ 23.9) was absent in **2**. The HMBC *J*^4^ correlation between the C-13 and the acetyl methyl indicates that the location of the acetyloxy group was at C-13 (Figure 2c). The ROESY correlation of H_2_-17/H_2_-12 revealed that OAc at C-13 was in *α*-orientation (Figure 2d). Moreover, the remaining chiral centers of **3** had a relative configuration identical to those of **1** based on their close similarity of the ^13^C NMR shifts and NOE data (Supplementary Material Figures S11–S20). Therefore, the structure of **2** was established as 5-angelate-13-acetyl-17-benzoyl-20-deoxyingenol (euphingenol B) (Figure 1).

The known compounds were identified as 17-*O*-benzoyl-20-deoxyingenol (**3**) [23], 3-angeloyloxy-ingena-1,6-dien-9-on-4*β*,5*β*-diol (**4**) [22], 5-angeloyloxy-ingena-1,6-dien-9-on-3*β*,4*β*-diol (**5**) [22], ingena-1,6-dien-9-on-3*β*,4*β*,5*β*,-triol (**6**) [22], 3-angeloyloxy-20-acetoxy-ingena-1,6-dien-9-on-4*β*,5*β*-diol (**7**) [25], ingenol-20-angelate (**8**) [26], 3-benzoyloxy-ingena-1,6-dien-9-on-4*β*,5*β*-diol (**9**) [27], ingenol-3-angelate (**10**) [28], 5-angeloyloxy-20-acetoxy-ingena-1,6-dien-9-on-3*β*-ol (**11**) [11], 3-angeloyloxy-6*β*,7*β*-epoxy-ingena-1-en-9-on-4*β*,5*β*-diol (**12**) [11], and 6*β*,7*β*-epoxy-3*β*,4*β*,5*β*-trihydroxyl-20-deoxyingenol (**13**) [29]. The identities of these compounds were established by comparing their MS and NMR spectroscopic data with those reported in the literature, and their structures are presented in Figure 1.

### 2.2. Bioactivity of the Compounds Towards Autophagic Flux

**Figure 3:**
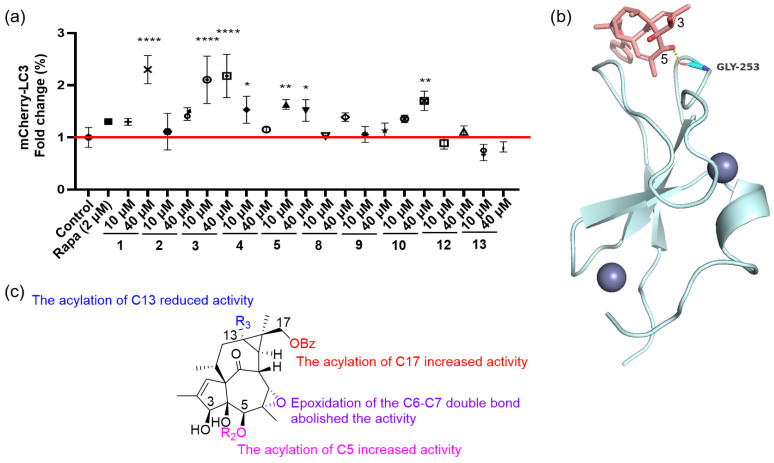
(**a**) The bioactivity of the compounds towards autophagic flux by flow cytometry analysis. Control refers to cells treated with dimethyl sulfoxide (DMSO). Rapamycin (Rapa, 2 μM) was used as the positive control. Three independent experiments were conducted, and the results are expressed as the mean ± SD (*n* = 3). Statistical significance was determined by one-way ANOVA followed by Dunnett’s multiple comparisons test. Results are considered to be statistically significant when *p* < 0.05. *, *p* < 0.05; **, *p* < 0.01; ****, *p* < 0.0001. Despite trace impurities in the ^1^H NMR spectra for some compounds, their impact on bioactivity was negligible given the lower test concentrations. (**b**) Molecular docking analysis for 17-*O*-benzoyl-20-deoxyingenol (**3**) on the C1 domain of PKC (PDB:7KO6) [39]. The O and N atoms are color-coded as red and blue, respectively. Compound **3** is color-coded as pink-orange. The PKC structure is shown as a cartoon, key residue GLY-253 is color-coded as blue sticks. Hydrogen bonds between compound **3** and the C1 domain are color-coded as yellow dashes. (**c**) Structure–activity relationship (SAR) analysis of ingenane diterpenes in the activation of autophagic flux.

To evaluate the effects of the isolated compounds on autophagic flux, flow cytometry analysis was performed using HM mCherry-GFP-LC3 cells—an autophagy reporter cell line established in our previous studies [7]. The compounds included euphingenol A (**1**), euphingenol B (**2**), 17-*O*-benzoyl-20-deoxyingenol (**3**), 3-angeloyloxy-ingena-1,6-dien-9-on-4*β*,5*β*-diol (**4**), 5-angeloyloxy-ingena-1,6-dien-9-on-3*β*,4*β*-diol (**5**), ingenol-20-angelate (**8**), 3-benzoyloxy-ingena-1,6-dien-9-on-4*β*,5*β*-diol (**9**), ingenol-3-angelate (**10**), 3-angeloyloxy-6*β*,7*β*-epoxy-ingena-1-en-9-on-4*β*,5*β*-diol (**12**), and 6*β*,7*β*-epoxy-3*β*,4*β*,5*β*-trihydroxyl-20-deoxyingenol (**13**). We used rapamycin (Rapa, an inducer of autophagy) as a positive control. Compared to the control (DMSO) and rapamycin (2 μM) treatment, significant activation of autophagic flux was observed upon treatment with compound **3** at concentrations of 10 μM and 40 μM (Figure 3a). Compound **1** also induced a dose-dependent increase in autophagic flux, with the 40 μM treatment showing comparable activity to compound **3**. Notably, the autophagic flux activation induced by compounds **1** (40 μM) and **3** (10 μM and 40 μM) was markedly higher than that of compound **5**, a compound previously reported by our group to regulate lysosomal biogenesis via dual pathways, direct PKC (protein kinase C) activation and parallel signaling, collectively enhancing lysosome formation [18]. Ingenane-type diterpenoids have been extensively studied for their diverse biological activities [30], including anti-inflammatory [31], anti-cancer [32,33], anti-HIV (human immunodeficiency virus) [34], anti-proliferative [35], multidrug resistance modulating [12,36], and anti-osteoporotic [37] effects. Notably, while certain ingenol esters have been reported to exhibit anti-adipogenic activity, compound **3** did not show such activity in the same study [23]. In addition, the present study reveals its potent activation of autophagic flux—a cellular process closely linked to the clearance of protein aggregates in neurodegenerative diseases.

A previous study reported that compound **5** promoted autophagy by binding to the PKCδ C1 domain [18]. Therefore, molecular docking was employed to further analyze the interaction of compound **3** with PKCδ. The docking results (Figure 3b) show that the oxygen atom of the hydroxyl group at C-5 can form a hydrogen bond with GLY253. In addition, insertion of the hydrophobic moiety of the ligand into the cell membrane is required for PKC activation [38]. The hydrophobic three membered ring, five membered ring, and two seven membered rings of the compound **3** skeleton are located on the surface of the binding domain, facilitating the insertion of the compound into the phospholipid bilayer. Therefore, we hypothesize that compound **3** may induce autophagy through binding to PKCδ. Crucially, our data imply that activator size significantly impacts efficacy: Compounds **1** and **2**, with coexisting angeloyl and C-17 benzoyl groups, exhibit reduced activation efficiency, potentially because excessive molecular bulk displaces PKC from the membrane, disrupting essential conformational changes. Conversely, compound **3** without the angeloyl moiety maintains optimal PKC-membrane proximity, enabling effective activation. These mechanistic hypotheses await experimental validation. Bryostatin is a marine-derived PKC modulator now in clinical trials for AD eradication [38]. Our results thus establish compound **3** as the most active compound in this series. Further exploration of compound **3** as a lead for autophagy-targeted therapeutic intervention in neurodegenerative conditions is strongly warranted.

Unlike compound **1**, which bears acyl modifications at multiple sites, compound **3** contains a benzoyl group exclusively at the C-17 position (Figure 3c). This observation further underscores the importance of C-17 acylation for enhancing autophagic flux, whereas Zhou et al. [32] reported that such modification diminishes PKC activation, suggesting distinct functional roles depending on the biological context. Furthermore, compound **2**, which carries an additional acetyl group at C-13 compared to compound **1**, did not exhibit significant activation of autophagic flux, suggesting that acylation at C-13 has no significant effect on the bioactivity. Notably, a discernible trend emerged from the structure–activity relationship (SAR) analysis: the oxidation of the C-6–C-7 double bond to an epoxide, as observed in compounds **12** and **13**, appeared to be detrimental to autophagic flux activation, leading to a complete loss of activity. These SAR observations are in agreement with those reported by Wu et al. [23], who also found that a benzoyloxy group at C-13 had minimal influence on activity, whereas epoxidation of the Δ^6^ double bond was unfavorable. Our findings highlight the critical role of site-specific acyl modifications in modulating the autophagy-inducing effects of these ingenane-type diterpenoids.

## 3. Materials and Methods

### 3.1. General Experiment

Melting points were measured using a Yuhua (Shanghai, China) X-4 digital microdisplay melting point apparatus. Optical rotation measurements were conducted with a Jasco P-1020 automatic polarimeter (Jasco, Tokyo, Japan). CD spectra were determined on an Applied Photophysics circular dichroism spectrometer (Applied Photophysics, Leatherhead, Surrey, UK). IR spectra were recorded on a NICOLET (Madison, WI, USA) iS107 mid-infrared spectrometer with KBr disks. NMR spectra were measured on a Bruker AVANCE III 500 MHz NMR spectrometer (Bruker, Billerica, MA, USA) with TMS (Sigma-Aldrich, St. Louis, MO, USA) as the internal standard. High-resolution MS data were recorded on an Agilent (Santa Clara, CA, USA) 1290 UPLC/6540 Q-TOF mass spectrometer in positive mode. An Agilent 1260 series instrument (Agilent Technologies, Santa Clara, CA, USA) equipped with a SunFire-C18 column (5 µm, 10 mm × 250 mm, Waters, Milford, MA, USA) and X Select HSS T3 (5 µm, 10 mm × 150 mm, Waters, Milford, MA, USA) was used for high-performance liquid chromatography (HPLC) semi-preparation. Fractions were monitored by thin-layer chromatography (TLC) on HSGF_254_ plates (Yantai Jiangyou Silica Gel Development Co., Ltd., Yantai, China), and spots were visualized by spraying with a vanillin chromogenic agent (Chongqi Chemical Co., Ltd., Chongqi, China). Silica gel (100–200, 200–300, 300–400) mesh (Qingdao Marine Chemical, Inc., Qingdao, China), MCI gel CHP 20P (75–150 μm, Mitsubishi Chemical Corporation, Tokyo, Japan), and Sephadex LH-20 gel (20–150 μm, Amersham Pharmacia Biotech AB, Uppsala, Sweden) were used for column chromatography (CC). TLC spots were visualized under UV light and by dipping into 5% H_2_SO_4_ in EtOH (Merck Millipore, Darmstadt, Germany), followed by heating. All solvent mixtures used for analyses and separations (HPLC and CC) are presented as the volume-to-volume ratio, unless otherwise specified.

### 3.2. Plant Materia

Whole plant parts of *E. peplus* were harvested in July 2020 from Kunming Botanical Garden, Yunnan Province, People’s Republic of China (coordinates: 102°44′ E, 25°07′ N, elevation ranging from 1800 to 2000 m above sea level). Taxonomic identification was performed by Prof. Hu Shi-Jun (Kunming Institute of Botany, Chinese Academy of Sciences). A representative voucher specimen (no. kep-09-13) has been deposited in the herbarium of the Kunming Institute of Botany, Chinese Academy of Sciences.

### 3.3. Extraction and Isolation

The air-dried and powdered whole plant material of *E. peplus* (60.0 kg) was extracted with methanol (3 × 40 L) at room temperature, each for 72 h. The combined methanol extracts were concentrated under reduced pressure to yield a crude residue (3.0 kg). This residue was then subjected to silica gel CC (100–200 mesh, 10 × 120 cm) and eluted with a gradient of petroleum ether/ethyl acetate (100:0 → 0:100, *v*/*v*) to yield four fractions (F1–F4) based on TLC analysis. Fraction F2 (800.3 g) was further separated by MCI gel CHP 20P CC (5 × 60 cm) using a stepwise gradient of MeOH–H_2_O (40:60 to 100:0) to yield six subfractions (F2-1–F2-6). Subfraction F2-5 (500.7 mg) was purified by silica gel CC (200–300 mesh, 3 × 40 cm) eluted with petroleum ether/ethyl acetate (20:1 to 1:1) to obtain F2-5-11 (290.2 mg). This fraction was further subjected to Sephadex LH-20 gel CC (20–150 μm, 2 × 150 cm) eluted with CH_2_Cl_2_/MeOH (50:50, *v*/*v*) to yield six subfractions (F2-5-11-1–F2-5-11-6). Subfraction F2-5-11-1 was purified by preparative HPLC using CH_3_CN/H_2_O (75:25, *v*/*v*) as the mobile phase at a flow rate of 3.0 mL/min to yield compounds **7** (15.0 mg) and **11** (7.2 mg). F2-5-11-2 was purified by semi-preparative HPLC (CH_3_CN/H_2_O, 70:30, *v*/*v*) to yield compounds **8** (12.5 mg) and **10** (2.3 mg). F2-5-11-3 was separated by preparative HPLC (CH_3_CN/H_2_O, 64:36, *v*/*v*) to yield compounds **2** (7.2 mg), **3** (13.6 mg), **4** (12.8 mg) and **5** (8.3 mg). F2-5-11-4 was successively purified by preparative HPLC (CH_3_CN/H_2_O, 65:35, *v*/*v*) to yield compounds **6** (4.7 mg), **12** (23.3 mg), **13** (24.7 mg). F2-5-11-5 was passed through preparative HPLC (CH_3_CN/H_2_O, 72:28, *v*/*v*) to yield compounds **1** (12.6 mg) and **9** (7.0 mg).

### 3.4. Compound Characterization

Euphingenol A (**1**): Yellowish amorphous powder; ${\left[\alpha \right]}_{D}^{24}$ +25.20 (*c* 0.15, MeOH); UV (MeOH) *λ*_max_ (log *ε*) 195 (4.57), 229 (3.97) nm; CD (MeOH): *λ* (∆*ε*) = 195 (−26.5), 204 (0.3), 224 (−2.8); IR (KBr) *ν*_max_ 3444, 1748, 1375, 1314, 1230, 1112, 713 cm^−1^; ^1^H and ^13^C NMR data; see Table 1; HRESIMS *m*/*z* 557.2510 [M + Na]^+^ (calcd. for C_32_H_38_O_7_Na, 557.2510).

Euphingenol B (**2**): Yellowish amorphous powder; ${\left[\alpha \right]}_{D}^{24}$ +40.45 (*c* 0.05, MeOH); UV (MeOH) *λ*_max_ (log *ε*) 195 (5.12), 227 (4.70) nm; CD (MeOH): *λ* (∆*ε*) = 196 (22.7), 224 (−3.0); IR (KBr) *ν*_max_ 2922, 1721, 1271, 1121, 713 cm^−1^; ^1^H and ^13^C NMR data; see Table 1; HRESIMS *m*/*z* 615.2566 [M + Na]^+^ (calcd. for C_34_H_40_O_9_Na, 615.2566).

### 3.5. Cell Culture and Bioactivity Assessment

A stable HM cell line harboring the mCherry-GFP-LC3 tripartite reporter construct was employed to evaluate the biological activities of compounds [6,7,40]. This chimeric protein comprises red fluorescent protein (mCherry), green fluorescent protein (GFP), and the autophagosome marker LC3, enabling direct visualization of autophagic flux dynamics. Under basal conditions, the co-localization of mCherry and GFP emissions generates yellow fluorescence in the cytosol. Upon autophagy induction, autophagosomes undergo fusion with lysosomes to generate autolysosomes; the acidic luminal pH selectively attenuates GFP fluorescence while leaving mCherry fluorescence intact, resulting in a predominant red signal that marks autolysosome formation [41,42]. An elevated red-to-green fluorescence ratio thus signifies efficient autophagosome-to-autolysosome transition. This method offers a significant advantage over conventional LC3-based assays, which cannot discern between accumulation of autophagosomes due to genuine induction versus blockade of downstream degradation. By enabling simultaneous visualization of both compartments, the mCherry-GFP-LC3 reporter system thus represents a reliable and sensitive tool for assessing the functional outcome of autophagic modulation by small molecules.

HM mCherry-GFP-LC3 cells were propagated in DMEM containing 10% fetal bovine serum (Gibco-BRL, 10099-141, Grand Island, NY, USA) and maintained at 37 °C in a humidified atmosphere containing 5% CO_2_ at 95% relative humidity. For experimental assays, cells in the logarithmic growth phase were seeded into 12-well plates at an appropriate density and allowed to adhere for 24 h prior to compound treatment. Test compounds were administered at final concentrations of 10 μM and 40 μM. Rapamycin (Rapa, 2 μM) (InvivoGen, San Diego, CA, USA) was used as a positive control. Following a 24 h incubation period, cells were collected and immediately fixed with 4% paraformaldehyde (PFA) (Sigma-Aldrich, St. Louis, MO, USA) for 15 min at room temperature. Fixed samples were subsequently subjected to flow cytometric analysis using a BD (Franklin Lakes, NJ, USA) FACSCanto II flow cytometer, and a total of 10,000 events were acquired per sample to quantify autophagic flux parameters, including the mCherry and GFP fluorescence intensities. All flow cytometry data were processed and analyzed using FlowJo software v11.1.1 (FLOWJO, LLC, Ashland, OR, USA). The ratio of red-to-green fluorescence was calculated to evaluate the progression of autophagic flux. Data analyses were carried out by using GraphPad Prism 8 (GraphPad Software, Inc., La Jolla, CA, USA). One-way ANOVA (analysis of variance) followed by Dunnett’s multiple comparisons test was performed to compare each treated group with the control group. Results are considered to be statistically significant when *p* < 0.05. *, *p* < 0.05; **, *p* < 0.01; ****, *p* < 0.0001.

### 3.6. Molecular Docking

The molecular docking study, including protein preparation, ligand preparation, and docking, was conducted using Schrödinger’s Maestro 12.9 (Schrödinger, Inc., New York, NY, USA). The X-ray crystal structure of the PKCδ C1 domain complex was obtained from the Protein Data Bank (PDB: 7KO6). PyMOL version 3.1.1 (Schrödinger, Inc., New York, NY, USA) was used to visualize the docking results.

## 4. Conclusions

A systematic phytochemical investigation of the medicinal plant *E. peplus* led to the isolation and characterization of thirteen ingenane-type diterpenoids. Among them, two previously undescribed compounds, euphingenol A and B (**1**–**2**), were identified together with 11 known analogs (**3**–**13**): 17-*O*-benzoyl-20-deoxyingenol (**3**), 3-angeloyloxy-ingena-1,6-dien-9-on-4*β*,5*β*-diol (**4**), 5-angeloyloxy-ingena-1,6-dien-9-on-3*β*,4*β*-diol (**5**), ingena-1,6-dien-9-on-3*β*,4*β*,5*β*,-triol (**6**), 3-angeloyloxy-20-acetoxy-ingena-1,6-dien-9-on-4*β*,5*β*-diol (**7**), ingenol-20-angelate (**8**), 3-benzoyloxy-ingena-1,6-dien-9-on-4*β*,5*β*-diol (**9**), ingenol-3-angelate (**10**), 5-angeloyloxy-20-acetoxy-ingena-1,6-dien-9-on-3*β*-ol (**11**), 3-angeloyloxy-6*β*,7*β*-epoxy-ingena-1-en-9-on-4*β*,5*β*-diol (**12**), and 6*β*,7*β*-epoxy-3*β*,4*β*,5*β*-trihydroxyl-20-deoxyingenol (**13**). The structures of the new compounds (**1**–**2**) were elucidated through comprehensive spectroscopic analyses, including HRESIMS and detailed 1D/2D NMR experiments (^1^H–^1^H COSY, HSQC, HMBC, and NOESY), which established their planar structures and relative configurations. This multi-technique approach represents the standard methodology for structure determination of complex natural products. The discovery of these two new ingenanes further enriches the chemical diversity reported for the genus Euphorbia and provides new materials for studying the biosynthetic pathways of this diterpenoid class in plants.

Biological evaluation revealed that compound **3** significantly activated autophagic flux in HM mCherry-GFP-LC3 cells at concentrations of 10 μM and 40 μM, whereas compound **1** induced a dose-dependent increase. Autophagy is a highly conserved degradative process in eukaryotic cells responsible for the clearance of misfolded proteins and damaged organelles, and its dysfunction is implicated in the pathogenesis of various neurodegenerative diseases, including AD. We hypothesize that compound **3** may induce autophagy by binding to PKCδ and may serve as a promising lead candidate for further development, pending in-depth mechanistic studies and in vivo evaluations to assess its potential relevance to neurodegenerative conditions. Notably, SAR analysis revealed that C-17 acylation enhances autophagic flux activation, while acylation at the C-13 position has no significant effect on bioactivity. This key finding provides a vital foundation for subsequent structure-guided chemical modifications aimed at optimizing the autophagy-inducing potency of ingenane-type diterpenoids.

## Figures and Tables

**Table 1:** ^1^H and ^13^C NMR Data of **1**–**2** in CDCl_3_ (*J* in Hz).

No.	1 ^a^		2 ^a^	
	*δ*_H_	*δ*_C_	*δ*_H_	*δ*_C_
1	5.97 d (1.5)	129.5	5.96 d (1.5)	128.8
2		139.6		140.0
3	3.73 brs	80.3	3.75 brs	80.2
4		85.2		85.0
5	5.26 s	76.8	5.27 s	76.0
6		135.3		135.8
7	5.85 dt (4.5, 1.8)	125.0	5.79 dt (4.6, 1.6)	123.9
8	4.42 ddt (12.1, 4.5, 1.8)	43.6	4.33 ddt (12.6, 4.6, 1.6)	43.2
9		206.1		205.3
10		72.7		72.7
11	2.46 overlapped	39.5	2.57 m	38.7
12a	2.46 overlapped	31.0	2.83 dd (16.9, 3.3)	35.3
12b	1.90 m		2.42 dd (16.9, 4.7)	
13	1.00 overlapped	23.9		68.7
14	1.21 dd (12.1, 8.3)	24.1	1.46 d (12.6)	28.8
15		27.9		34.2
16a	4.59 d (12.0)	66.3	4.59 d (12.1)	65.8
16b	4.48 d (12.0)		4.45 d (12.1)	
17	1.22 s	24.6	1.26 s	18.7
18	1.00 overlapped	17.1	1.01 d (7.2)	18.2
19	1.83 d (1.5)	15.5	1.83 d (1.1)	15.5
20	1.51 s	21.4	1.48 s	21.4
5-OAng		167.1		167.1
2′		127.0		126.9
3′	6.16 m	140.2	6.16 m	140.5
4′	1.98 d (7.3)	16.0	1.98 d (7.3)	16.0
5′	1.94 m	20.7	1.93 m	20.7
13-OAc				170.8
			2.01 s	21.2
17-OBz		166.9		166.8
2″		130.4		130.2
3″, 7″	8.05 d (8.4)	129.6	8.14 d (8.4)	129.8
4″, 6″	7.45 t (7.8)	128.4	7.46 t (7.8)	128.4
5″	7.56 t (7.4)	133.0	7.56 t (7.4)	133.0

^a^ Recorded in CDCl_3_ at 500 MHz (^1^H) and 125 MHz (^13^C). S, singlet; d, doublet; t, triplet; dd, doublet of doublets; dt, doublet of triplets; ddt, doublet of doublets of triplets; brs, broad singlet; m, multiplet; overlapped, overlapped signal.

## Data Availability

The data supporting the findings of this article are available from the author, J.W., upon reasonable request.

## Supplementary Materials

The following supporting information can be downloaded at: https://www.mdpi.com/article/10.3390/molecules31091388/s1, Figures S1–S10: Spectral data (^1^H NMR, ^13^C NMR, ^1^H-^1^HCOSY, HSQC, HMBC, ROESY, CD, UV, IR and (+)-HRESI-MS) for compound **1** (euphingenol A); Figures S11–S20: Spectral data (^1^H NMR, ^13^C NMR, ^1^H-^1^HCOSY, HSQC, HMBC, ROESY, CD, UV, IR and (+)-HRESI-MS) for compound **2** (euphingenol B).

## Abbreviations

The following abbreviations are used in this manuscript:

AD	Alzheimer’s disease
ALP	Autophagy–lysosomal pathway
FDA	Food and drug administration
HRESIMS	High-resolution electrospray ionization mass spectroscopy
NMR	Nuclear magnetic resonance
CD	Circular dichroism
UV	Ultraviolet–visible spectroscopy
IR	Infrared spectroscopy
NOE	Nuclear Overhauser effect
DMEM	Dulbecco’s modified eagle medium
