# Supplementary material for: Ingenane Diterpenoids from Euphorbia peplus: Structure Elucidation and Autophagic Flux Activation Activity
Source: Molecules. 2026 Apr 23;31(9):1388. doi: 10.3390/molecules31091388 (PMC13165047; doi:10.3390/molecules31091388)
Supplement: Supplementary file 1 [file molecules-31-01388-s001.zip › molecules-4215999-supplementary.pdf]

Supporting Information

Ingenane Diterpenoids from *Euphorbia peplus*: Structure Elucidation and Autophagic Flux Activation Activity

Jiajia Wan [1,2], Qingyun Lu [1,2], Zifei Xu [1], Xiaojiang Hao [1,3], Rongcan Luo [4,*] and Yingtong Di [1,*]

[1] State Key Laboratory of Phytochemistry and Plant Resources in West China, Kunming Institute of Botany, Chinese Academy of Sciences, Kunming 650201, China; wanjiajia@mail.kib.ac.cn (J.W.); m19184437531@163.com (Q.L.); xuzifei@mail.kib.ac.cn (Z.X.); haoxj@mail.kib.ac.cn (X.H.)

[2] University of Chinese Academy of Sciences, Beijing 100049, China

[3] Yunnan Characteristic Plant Extraction Laboratory, Kunming, 650106, China

[4] Gansu Key Laboratory of Biomonitoring and Bioremediation for Environmental Pollution, Ministry of Education Key Laboratory of Cell Activities and Stress Adaptations, School of Life Sciences, Lanzhou University, Lanzhou 730099, China

* Correspondence: luorc@lzu.edu.cn (R.L.); diyt@mail.kib.ac.cn (Y.D.)

List of Contents

Figure S1. ^{1}H NMR spectrum of euphingenol A 3

Figure S2. ^{13}C NMR spectrum of euphingenol A 3

Figure S3. ^{1}H-^{1}H COSY spectrum of euphingenol A 4

Figure S4. HSQC spectrum of euphingenol A 4

Figure S5. HMBC spectrum of euphingenol A 5

Figure S6. ROESY spectrum of euphingenol A 5

Figure S7. CD spectrum of euphingenol A 6

Figure S8. UV spectrum of euphingenol A 6

Figure S9. IR spectrum of euphingenol A 7

Figure S10. (+)-HRESIMS spectrum of euphingenol A 7

Figure S11. ^{1}H NMR spectrum of euphingenol B 8

Figure S12. ^{13}C NMR spectrum of euphingenol B 8

Figure S13. ^{1}H-^{1}H COSY spectrum of euphingenol B 9

Figure S14. HSQC spectrum of euphingenol B 9

Figure S15. HMBC spectrum of euphingenol B 10

Figure S16. ROESY spectrum of euphingenol B 10

Figure S17. CD spectrum of euphingenol B 11

Figure S18. UV spectrum of euphingenol B 11

Figure S19. IR spectrum of euphingenol B 12

Figure S20. (+)-HRESIMS spectrum of euphingenol B 12

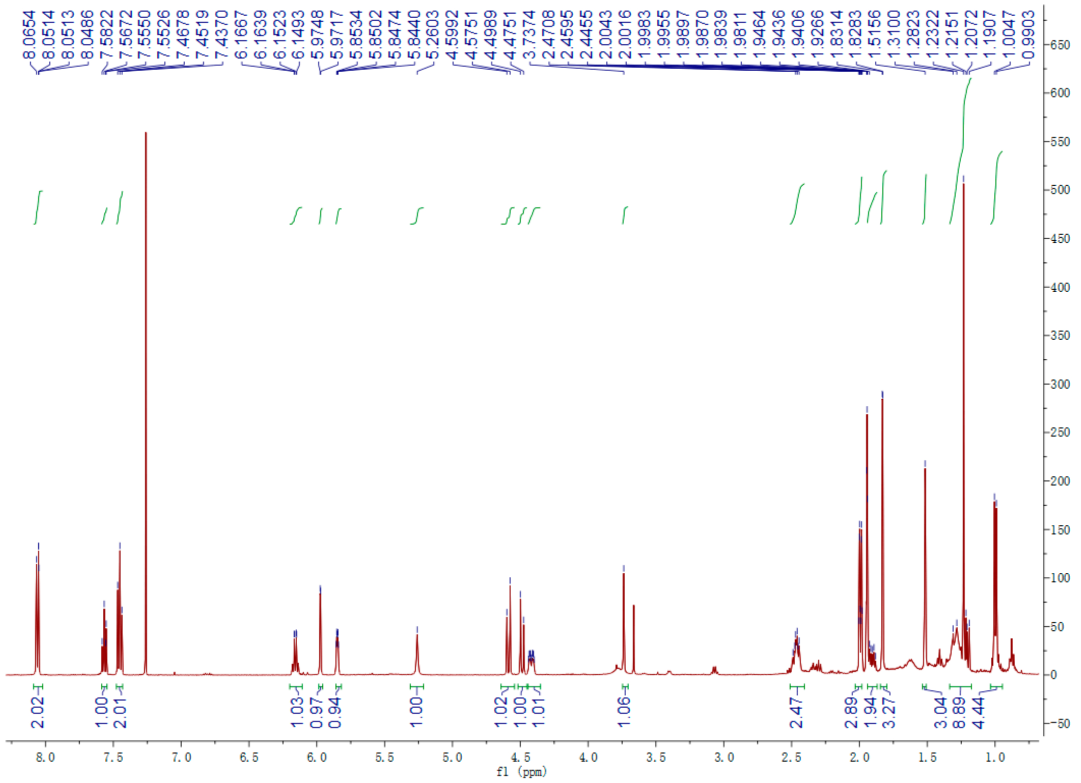

Figure S1. ^{1}**H-NMR spectrum of euphingenol A.**

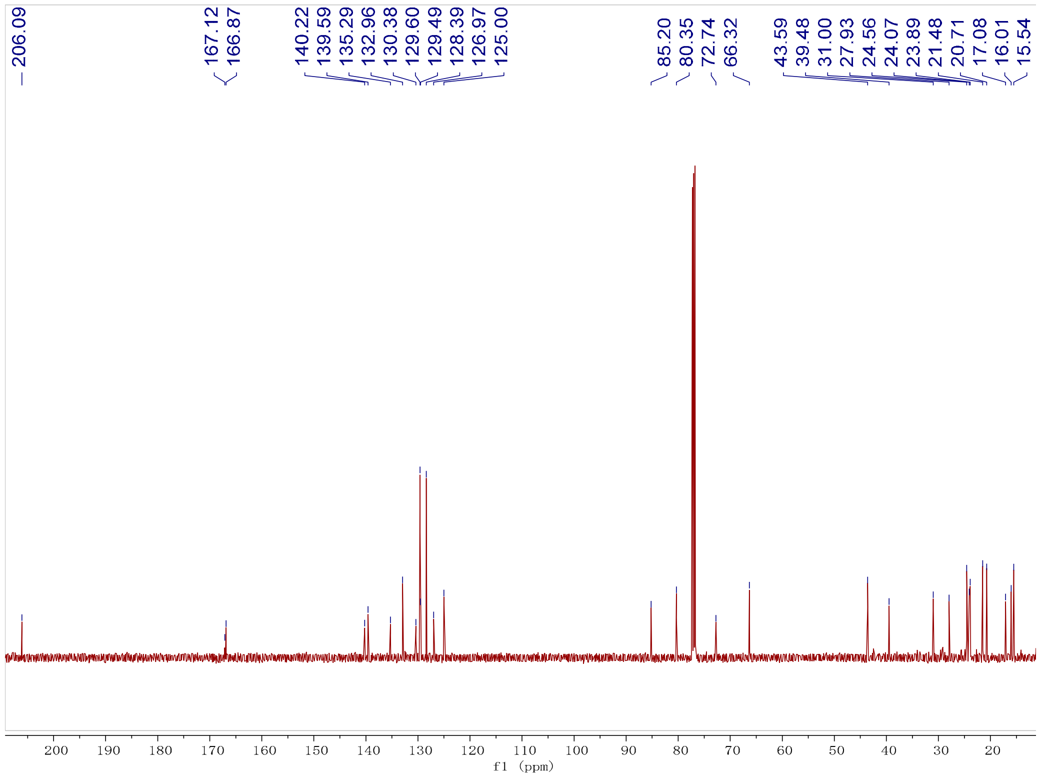

Figure S2. ^{13}**C-NMR spectrum of euphingenol A.**

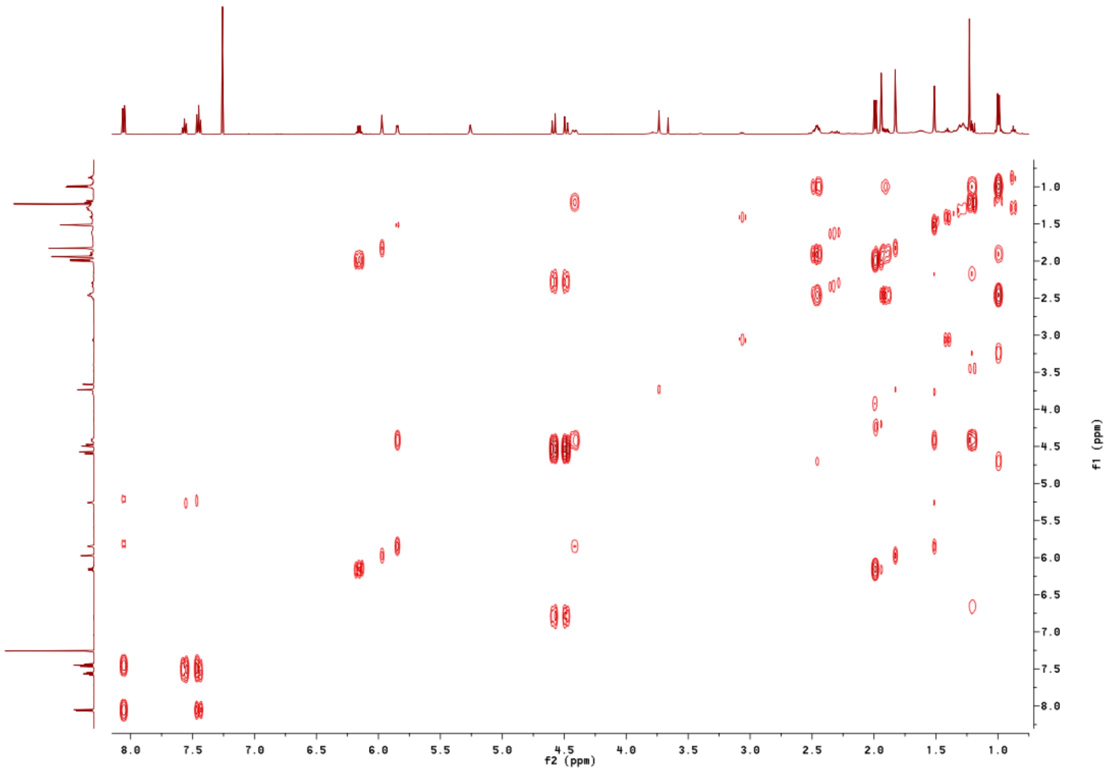

Figure S3. COSY spectrum of euphingenol A.

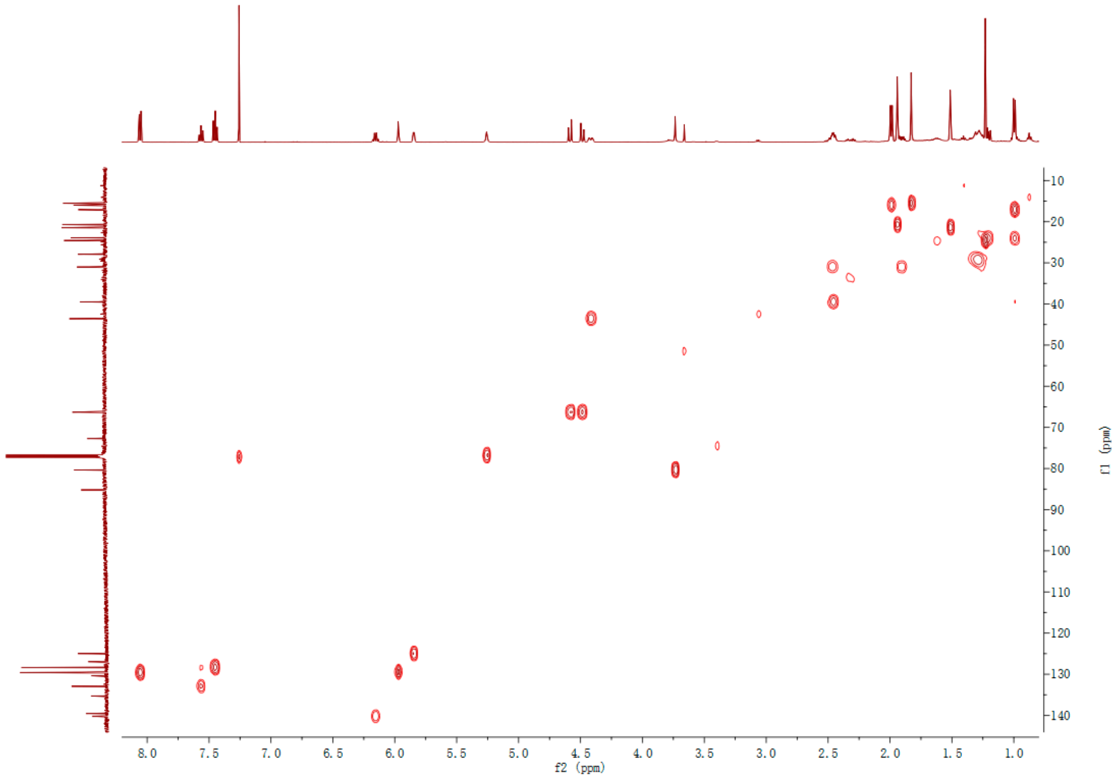

Figure S4. HSQC spectrum of euphingenol A.

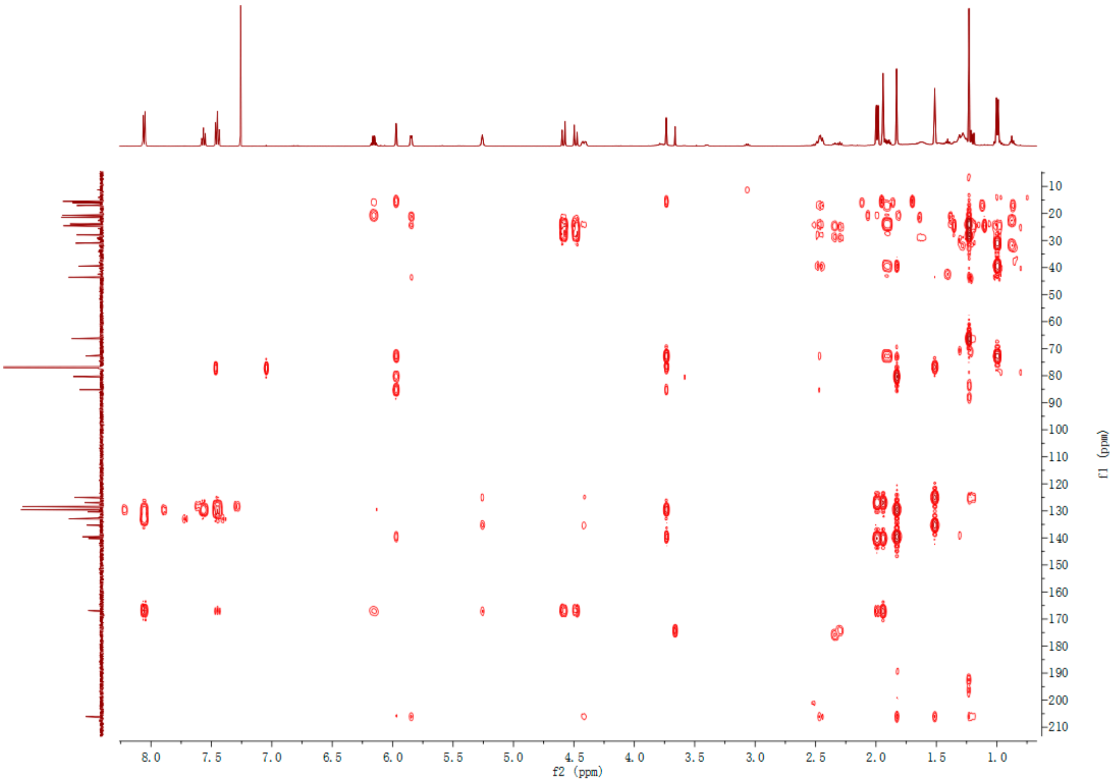

Figure S5. HMBC spectrum of euphingenol A.

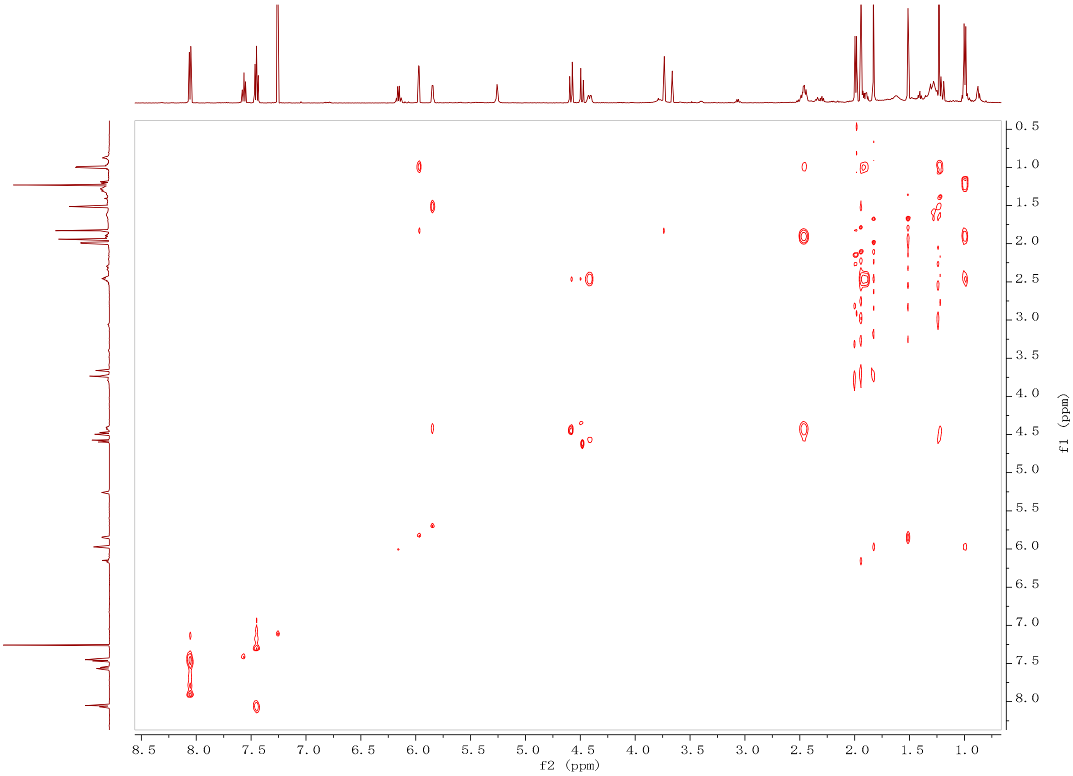

Figure S6. ROESY spectrum of euphingenol A.

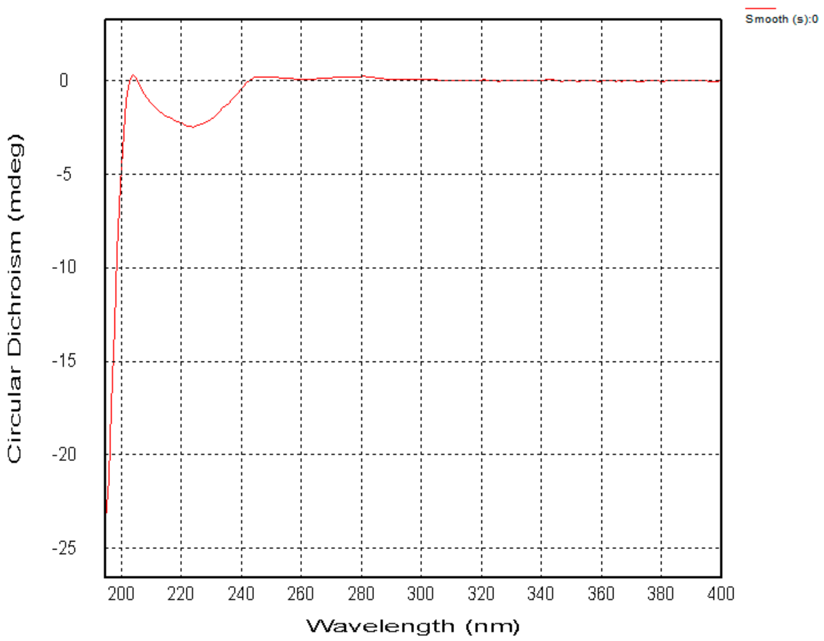

Figure S7. CD spectrum of euphingenol A compound 1.

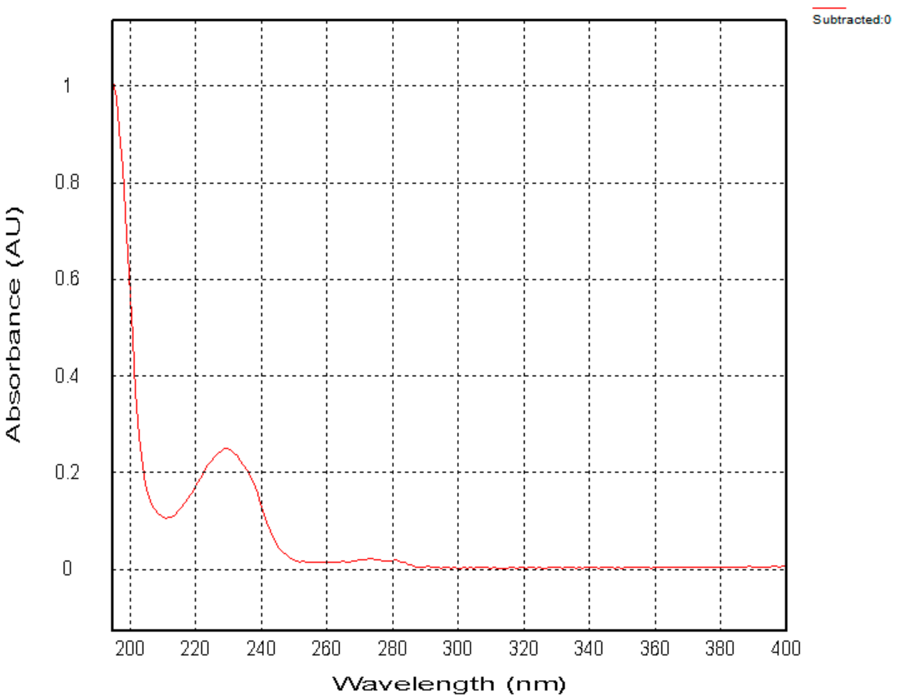

Figure S8. UV spectrum of euphingenol A.

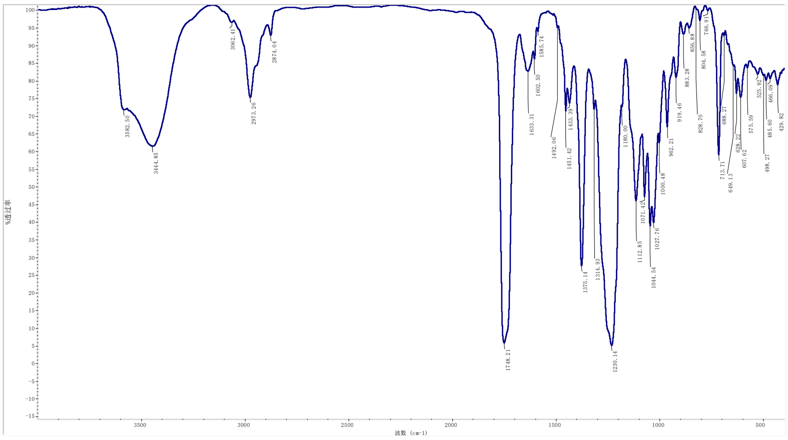

Sample Name: HLQY-562
KBr压片
采集时间: 星期二 8月 25 16:33:10 2020 (GMT+08:00)
仪器型号: NICOLET iS10
Software version: OMNIC 9.8.372

样品扫描次数: 16
背景扫描次数: 16
分辨率: 4.000
采样增益: 1.0
动镜速度: 0.4747
光阑: 80.00

Figure S9. IR spectrum of euphingenol A.

Qualitative Analysis Report

**Data Filename**	HLQY-541.d	**Sample Name**	HLQY-541
**Sample Type**	Sample	**Position**	P1-A1
**Instrument Name**	Instrument 1	**User Name**	
**Acq Method**	s.m	**Acquired Time**	5/8/2020 10:06:05 AM
**IRM Calibration Status**	Success	**DA Method**	Default.m
**Comment**			

**Sample Group**		**Info.**
**Acquisition SW**	6200 series TOF/6500 series	
**Version**	Q-TOF B.05.01 (B5125.2)	

User Spectra

Fragmentor Voltage	Collision Energy	Ionization Mode
135	0	ESI

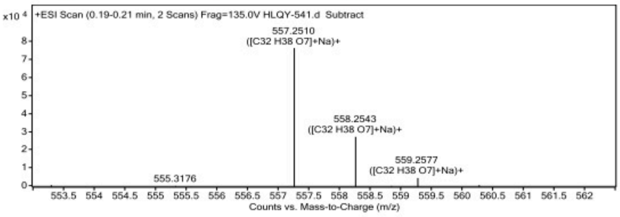

Peak List

m/z	z	Abund	Formula	Ion
274.2741	1	2341.33		
318.3009	1	6101.73		
362.3253	1	2278.79		
435.2172	1	4396.7		
557.251	1	76667.83	C32 H38 O7	(M+Na)+
558.2543	1	27732.42	C32 H38 O7	(M+Na)+
559.2577	1	4988.2	C32 H38 O7	(M+Na)+
573.2266	1	3022.67		
580.3272	1	9894.86		
581.3309	1	3828.16		

Formula Calculator Element Limits

Element	Min	Max
C	3	60
H	0	120
O	0	30

Formula Calculator Results

Formula	CalculatedMass	CalculatedMz	Mz	Diff. (mDa)	Diff. (ppm)	DBE
C32 H38 O7	534.2618	557.2510	557.2510	0.00	0.00	14.0000

Figure S10. HR-ESI-MS spectrum of euphingenol A.

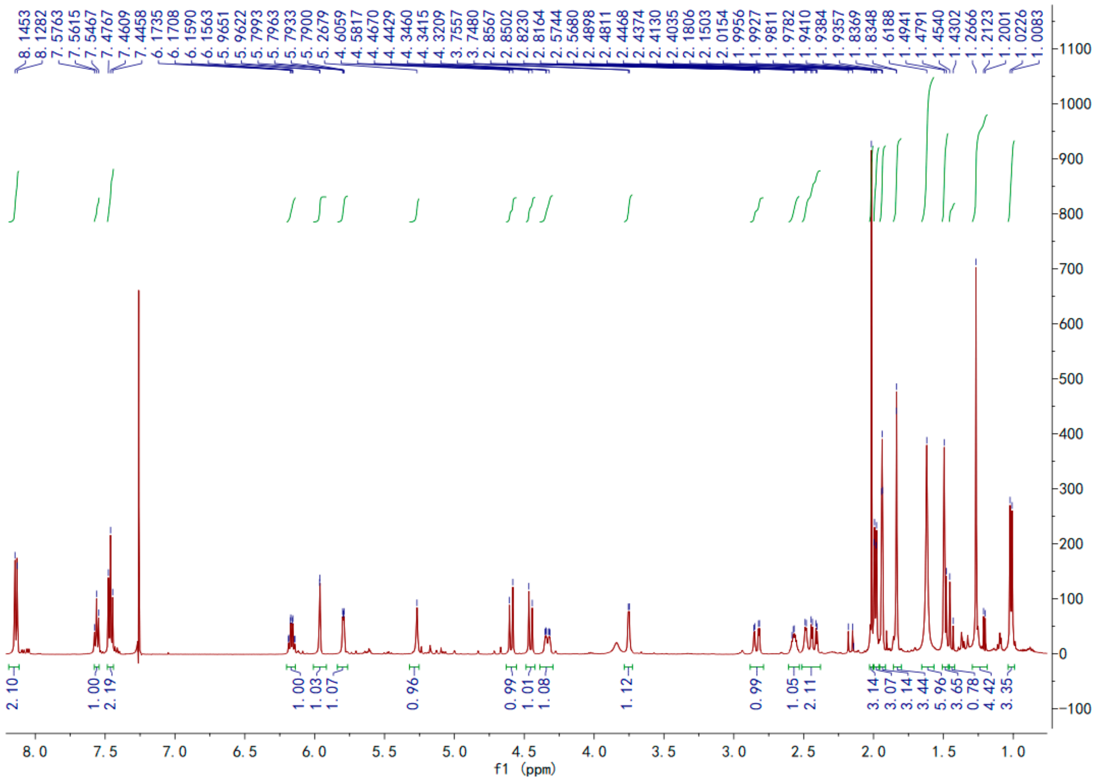

Figure S11. ^{1}**H-NMR spectrum of euphingenol B.**

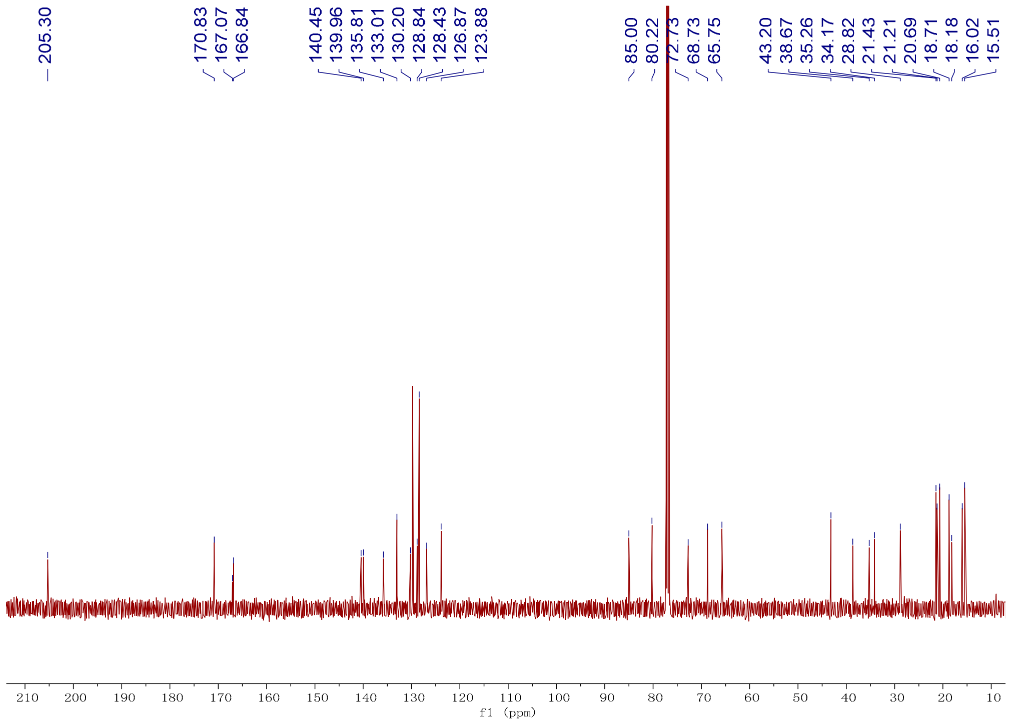

Figure S12. ^{13}**C-NMR spectrum of euphingenol B.**

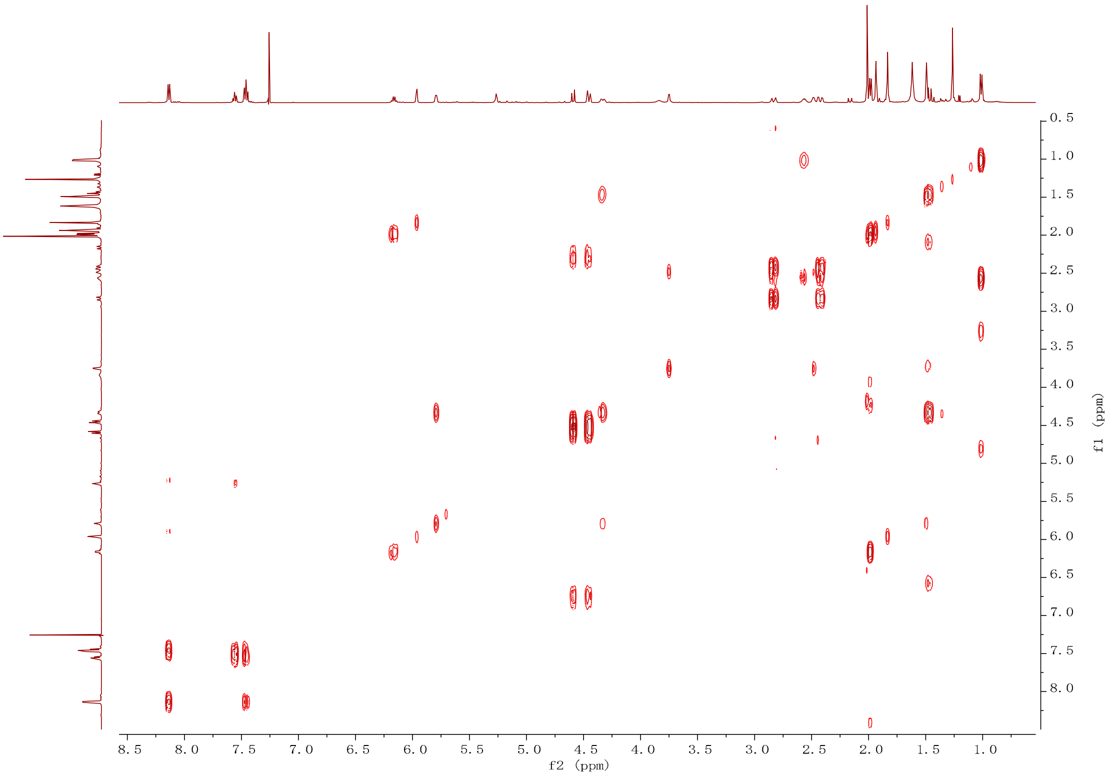

Figure S13. COSY spectrum of euphingenol B.

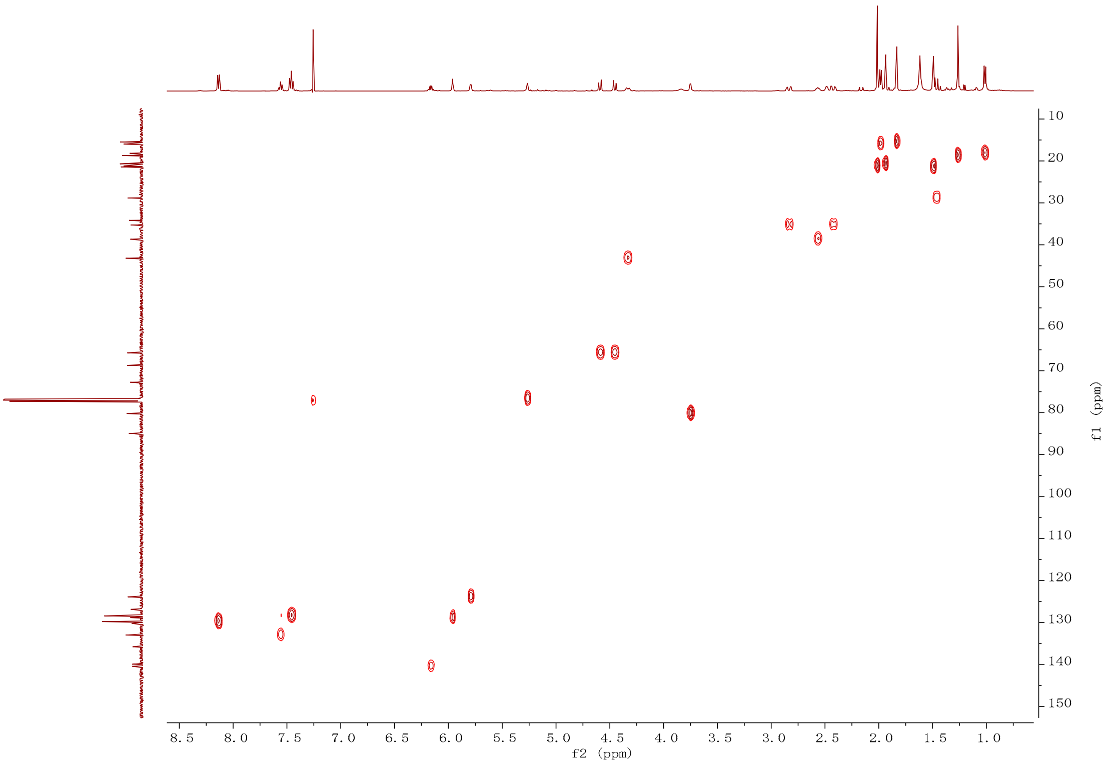

Figure S14. HSQC spectrum of euphingenol B.

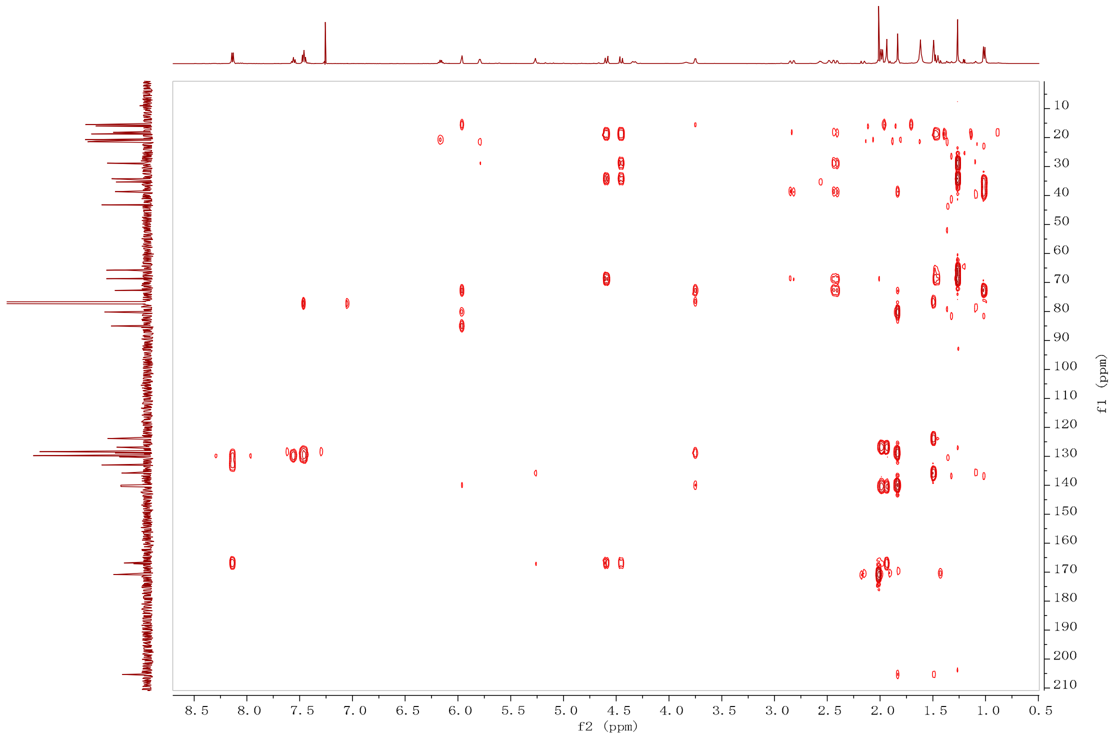

Figure S15. HMBC spectrum of euphingenol B.

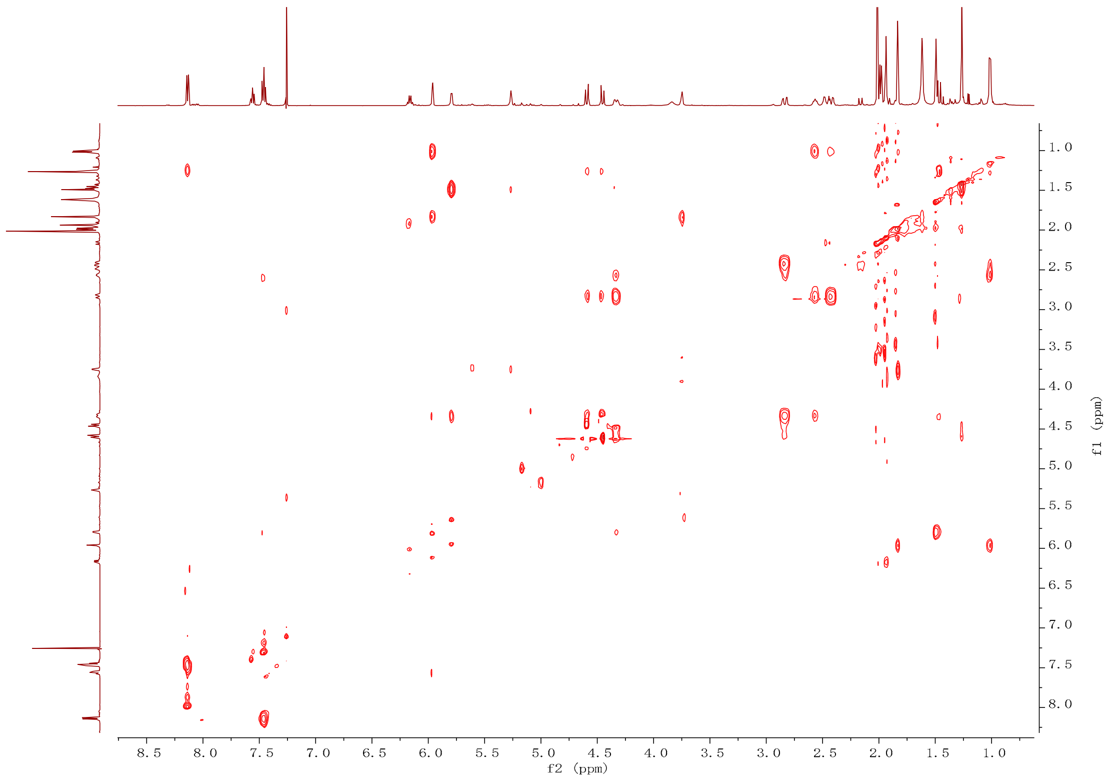

Figure S16. ROESY spectrum of euphingenol B.

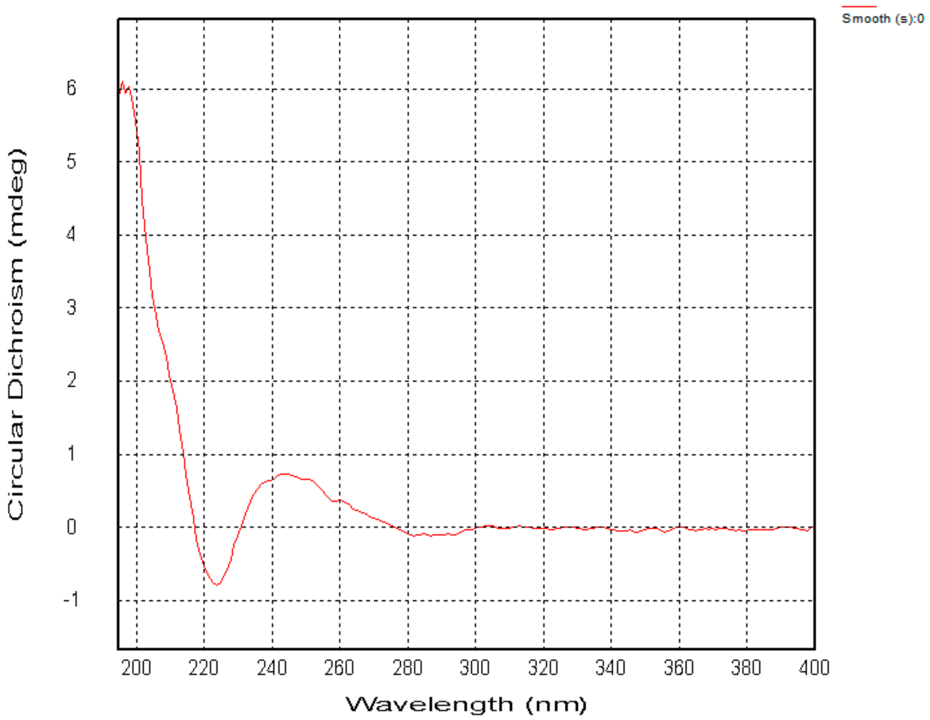

Figure S17. CD spectrum of euphingenol B.

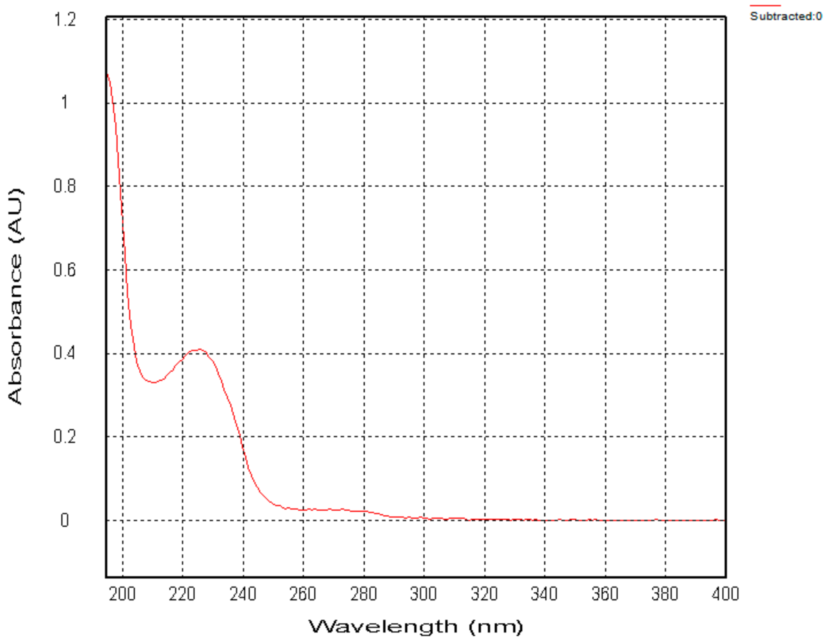

Figure S18. UV spectrum of euphingenol B.

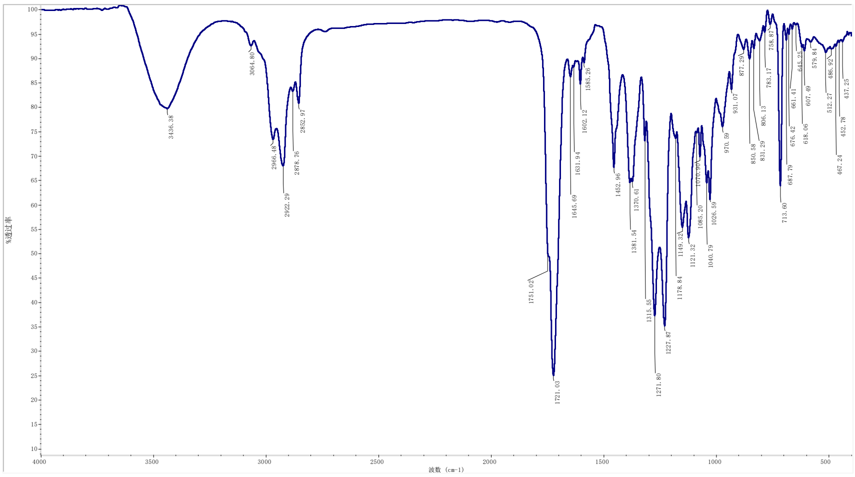

Sample Name: HLQY-341
KBr压片
采集时间: 星期一 8月 24 16:19:25 2020 (GMT+08:00)
仪器型号: NICOLET iS10
Software version: OMNIC 9.8.372

样品扫描次数: 16
背景扫描次数: 16
分辨率: 4.000
采样增益: 1.0
动镜速度: 0.4747
光阑: 80.00

Figure S19. IR spectrum of euphingenol B.

Qualitative Analysis Report

**Data Filename**	HLQY-341.d	**Sample Name**	HLQY-341
**Sample Type**	Sample	**Position**	P1-C5
**Instrument Name**	Instrument 1	**User Name**	
**Acq Method**	s.m	**Acquired Time**	7/13/2020 3:11:34 PM
**IRM Calibration Status**	Success	**DA Method**	Default.m
**Comment**			

**Sample Group**		**Info.**
**Acquisition SW**	6200 series TOF/6500 series	
**Version**	Q-TOF B.05.01 (B5125.2)	

User Spectra

Fragmentor Voltage	Collision Energy	Ionization Mode
135	0	ESI

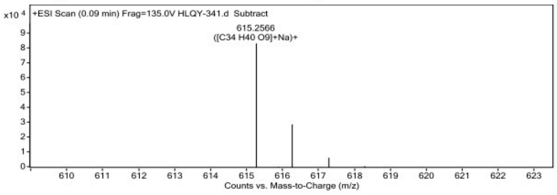

Peak List

m/z	z	Abund	Formula	Ion
615.2566	1	83478.45	C34 H40 O9	(M+Na)+
616.2603	1	29055.01	C34 H40 O9	(M+Na)+
617.2631	1	6642.15	C34 H40 O9	(M+Na)+
638.3326	1	14997.23		
665.2932	1	21730.42		
666.298	1	8494.76		
756.2992	1	11918.95		
1207.5277	1	41747.72		
1208.5314	1	33508.94		
1209.5348	1	13218.26		

Formula Calculator Element Limits

Element	Min	Max
C	3	60
H	0	120
O	0	30

Formula Calculator Results

Formula	CalculatedMass	CalculatedMz	Mz	Diff. (mDa)	Diff. (ppm)	DBE
C34 H40 O9	592.2672	615.2565	615.2566	-0.10	-0.16	15.0000

Figure S20. HR-ESI-MS spectrum of euphingenol B.
